# Pre-operative stereotactic radiosurgery versus intraoperative cesium-131 brachytherapy for resected brain metastases: a systematic review and meta-analysis

**DOI:** 10.1007/s11060-026-05870-3

**Published:** 2026-11-09

**Authors:** Trent Kite, Danny A. Mahmoudi, Eric Singh, Henry Knox, Bhavika Gupta, Alexander C. Whiting, Stephen Karlovits, Rodney E. Wegner, Brian Williams, Matthew J. Shepard

**Affiliations:** 1 Department of Neurosurgery, Allegheny Health Network Neuroscience Institute, 320 E North Ave, Pittsburgh, PA 15212 USA; 2 Drexel University College of Medicine, Philadelphia, PA USA; 3 Edward Via College of Osteopathic Medicine, Carolinas Campus, Spartanburg, SC USA; 4 Department of Radiation Oncology, Allegheny Health Network Cancer Institute, Pittsburgh, PA USA; 5 Department of Neurosurgery, University of Louisville, Louisville, KY USA

**Keywords:** Stereotactic radiosurgery, Brachytherapy, Cesium-131, Pre-operative stereotactic radiosurgery, Brain metastases

## Abstract

**Background:**

Pre-operative stereotactic radiosurgery (SRS) and intraoperative cesium-131 (Cs-131) brachytherapy are alternatives to post-operative SRS for resected brain metastases (BMs). The randomized phase 3 Radiation One and Done Study (ROADS; NCT04365374) showed improved surgical-bed control with Cs-131 tiles versus post-operative stereotactic radiotherapy, but outcomes relative to pre-operative SRS are undefined.

**Methods:**

PubMed, Embase, and ScienceDirect were searched through October 2026 (PRISMA 2020); one further trial was identified by other methods. Twelve-month local control (LC), leptomeningeal disease (LMD), radiation necrosis (RN), and overall survival (OS) were extracted for treatment-naïve BMs. Proportions were pooled with random-effects generalized linear mixed models and compared with a test for subgroup differences; sensitivity analyses included meta-regression, Hartung–Knapp, leave-one-out, follow-up, and fractionation analyses.

**Results:**

Fifteen pre-operative SRS studies (918 patients/940 BMs) and four brachytherapy studies (207 patients/217 BMs) were included. Pooled 12-month LC was 97% (95% CI 90–99) with Cs-131 brachytherapy and 90% (86–93) with pre-operative SRS (test for subgroup differences *p* = 0.054; meta-regression *p* = 0.019; Hartung–Knapp *p* = 0.066); among studies reporting observed counts, LC was 99% vs. 90% (*p* = 0.009). Within pre-operative SRS, LC was 96% after hypofractionated and 86% after single-fraction treatment (*p* = 0.005). LMD (9.1% vs. 5.1%) and RN (0.7% vs. 5.2%) did not differ significantly. Median OS was 15.6 months with brachytherapy (range 5.9–42.5; 11.0 months, range 5.9–20.1, excluding the ROADS estimate) and 14.4 months with pre-operative SRS (range 6.3–26.4).

**Conclusion:**

Both approaches achieved high LC with low rates of LMD and RN. There was a trend towards higher LC with Cs-131 brachytherapy than with pre-operative SRS (97% vs. 90%; *p* = 0.054), and significantly so among studies reporting observed counts (99% vs. 90%; *p* = 0.009). Hypofractionated pre-operative SRS achieved LC similar to that of brachytherapy, suggesting that the difference may reflect single-fraction dose de-escalation rather than an intrinsic advantage of either modality. The two techniques may serve complementary roles, and prospective comparative study is warranted.

**Registration:**

This review was not registered in PROSPERO.

**Supplementary Information:**

The online version contains supplementary material available at https://doi.org/10.1007/s11060-026-05870-3.

## Introduction

Brain metastases (BMs) are associated with substantial morbidity and mortality and require prompt, effective management [1]. Adjuvant whole-brain radiotherapy (WBRT) after resection improved intracranial control in the trial by Patchell et al. [2] but its neurocognitive toxicity, together with advances in radiation delivery, led to the adoption of post-operative stereotactic radiosurgery (SRS) as the preferred adjuvant modality [3–7]. Post-operative SRS nonetheless has important limitations, including delays to adjuvant treatment, dose de-escalation for large cavities, uncertainty in cavity target delineation, and leptomeningeal disease (LMD) after resection [8–13].

Pre-operative SRS treats the intact metastasis, which improves target delineation and may reduce the dissemination of viable tumor cells into the cerebrospinal fluid (CSF) at resection [14, 15]. However, pre-operative targets are often large and may require dose reduction or hypofractionation, and the approach is generally not feasible for patients who require urgent surgical decompression [14, 16]. Intracavitary cesium-131 (Cs-131) brachytherapy, placed at the time of resection, begins adjuvant radiation immediately and avoids a separate post-operative course [17–19]. In the randomized phase 3 Radiation One and Done Study (ROADS), collagen-embedded Cs-131 tiles (GammaTile) improved surgical-bed recurrence-free survival compared with post-operative stereotactic radiotherapy (HR 0.48, *p* = 0.002). LMD was numerically higher with brachytherapy (9.7% vs. 3.0%), although the difference was not statistically significant (*p* = 0.15), and radiation necrosis (RN) was similar (5.3% vs. 5.7%) [20].

Pre-operative SRS and Cs-131 brachytherapy both address limitations of post-operative SRS, but they have not been compared. We therefore performed a systematic review and meta-analysis of pre-operative SRS and Cs-131 brachytherapy for resected, treatment-naïve BMs. The primary endpoint was 12-month local control (LC); secondary endpoints were LMD, RN, and overall survival (OS).

## Methods

### Protocol and eligibility criteria

This review followed the PRISMA 2020 statement (checklist, Supplementary Table S5) [21]. The protocol was not prospectively registered. Studies of pre-operative SRS or intracavitary Cs-131 brachytherapy for newly diagnosed, treatment-naïve BMs in adults with a histologically confirmed extracranial primary malignancy were eligible. Studies were excluded if they involved prior resection or radiation to the treated lesion, follow-up shorter than 3 months (unless death occurred earlier), non-metastatic pathology, other intraoperative radiotherapy techniques, or LMD at the time of treatment. Cohorts that mix newly diagnosed and recurrent lesions were included only when outcomes for the newly diagnosed lesions were reported separately at the group level. Non-English studies, case reports, conference abstracts, reviews, and duplicate cohorts were also excluded.

### Search strategy

PubMed/MEDLINE, Embase, and ScienceDirect were searched from inception through 1 October 2026 (PubMed/MEDLINE and Embase) and 30 October 2026 (ScienceDirect), and the reference lists of included articles were hand-searched. One further study, the STEP trial [22], was identified by following up a trial protocol retrieved by the database search; it was published after the search date. Separate strategies were used for pre-operative SRS and brachytherapy, each combining intervention and brain metastasis terms, with an additional pre-operative timing block for the SRS search. Searches were limited to English-language articles, with the research-article filter applied in ScienceDirect. Full search strings for each database are provided in Supplementary Table S1.

### Study selection and data extraction

Four reviewers (D.M., E.S., T.K., H.K.) screened titles, abstracts, and full texts in Rayyan, resolving discrepancies by consensus. Full-text reports were excluded if they did not meet the eligibility criteria above. When studies reported overlapping patients, one non-overlapping set of cohorts was retained: the PROPS-BM multicenter cohort [23] served as the pre-operative SRS anchor; the INTERNEO pooled analysis [24] was replaced by its non-overlapping sites; [25–28] and reports subsumed within either were excluded [15, 29, 30]. Wernicke et al. (2017) treated 46 lesions, 8 of which were salvage treatments of recurrent, previously irradiated lesions; [31] only the 38 treatment-naïve lesions, which were reported separately, were analyzed. Three reviewers (T.K., D.M., H.K.) extracted data independently, with final verification by T.K.; for the five studies added during revision, each extracted value was linked to its source page and verified against the full text by D.M. Extracted variables included study design, numbers of patients and lesions, age, Karnofsky Performance Status (KPS), extracranial disease, systemic therapy, histology, tumor location and volume, platform, dose, fractionation, isodose line, margin, recurrence definition, and outcomes.

### Outcomes and derivation of event counts

The primary endpoint was 12-month LC. This time point was chosen because it is the conventional endpoint in studies of cavity radiosurgery and was the only LC time point reported by nearly all included studies; follow-up was insufficient to support later time points. Secondary endpoints were LMD, RN, and OS. Twelve-month LC counts were obtained in one of two ways (Supplementary Table S4): observed counts reported directly (Hoyle, Kutuk, Marwaha, Rogers, Schröder, Weinberg, Wernicke), in which events were counted over follow-up and denominators include patients not followed to 12 months; or counts reconstructed by multiplying a published 12-month Kaplan–Meier or cumulative-incidence estimate by the analyzed sample size (Agrawal, Biau, Deguchi, Higazy, Jansen, Li, Mallela, Prabhu, Yaprak, Kite, Mahapatra). Reconstructed counts treat all patients as followed to 12 months and therefore overstate precision. LMD and RN were extracted as crude proportions unless only a 12-month estimate was available. Counts were lesion-level where reported and patient-level otherwise (PROPS-BM, Schröder, and Wernicke were lesion-level; Agrawal, Kutuk, and Kite reported only patients despite multiple lesions; in the remaining studies each patient had one lesion); LMD in PROPS-BM was patient-level. Median OS was extracted as reported.

### Risk of bias

Two reviewers (E.S., D.M.) independently assessed methodological quality from the full-text articles using the eight non-comparative items of the Methodological Index for Non-Randomized Studies (MINORS; maximum score 16), with disagreements resolved by consensus [32]. ROADS is a randomized trial; because only its Cs-131 arm was used, it was scored as a single-arm cohort with the same non-comparative items for consistency. Scores of ≥ 13, 9–12, and ≤ 8 were considered low, moderate, and high risk of bias, respectively. Item-specific scoring rules are provided in Supplementary Table S2.

### Statistical analysis

Proportions were pooled on the logit scale with a binomial-normal generalized linear mixed model (GLMM; maximum likelihood), which models event counts directly and requires no continuity correction for proportions of 0% or 100% [33]. Each modality was pooled with its own between-study variance (τ²). The primary comparison between modalities was the test for subgroup differences, which compares the two separately estimated pooled proportions and allows heterogeneity to differ between modalities. This test was preferred to mixed-effects meta-regression because between-study variance was larger among the four brachytherapy studies than among the pre-operative SRS studies, and a meta-regression with a single shared τ² would understate the uncertainty of the brachytherapy estimate. Meta-regression (modality as moderator, shared τ²) and inverse-variance logit pooling with Hartung–Knapp adjustment [34] were reported as secondary analyses (Supplementary Figures S1–S3); the Hartung–Knapp interval was not allowed to be narrower than the standard random-effects interval. Heterogeneity was summarized with τ² and I².

Sensitivity analyses for LC excluded ROADS (the largest Cs-131 cohort, industry-sponsored, and co-authored by a study author) and PROPS-BM (49% of pre-operative SRS LC observations, registry-based with local outcome assessment), and excluded each study in turn (leave-one-out), including STEP (published after the search date), Kite et al. (co-authored by study authors; 36 of 51 patients censored before 12 months), Wernicke et al. (2017), and Mahapatra et al. (median follow-up 2.9 months). Further analyses used the whole Wernicke et al. cohort in place of its treatment-naïve stratum; were restricted to studies with median follow-up of at least 12 months or all patients followed for at least 1 year, to studies with observed counts, and to studies with patient-level denominators; and replaced rate-derived counts with crude counts where both were reported. Within pre-operative SRS, LC was compared between single-fraction and hypofractionated treatment among studies using one regimen, and again with mixed-schedule studies counted as single-fraction. A pooled estimate across modalities was not calculated. Publication bias was not assessed because only four brachytherapy studies contributed to the comparison. Median OS was summarized as the median of study-level medians and was not tested. Analyses used the meta and metafor packages in R version 4.5.3 [35, 36].

## Results

### Study selection

**Fig. 1:**
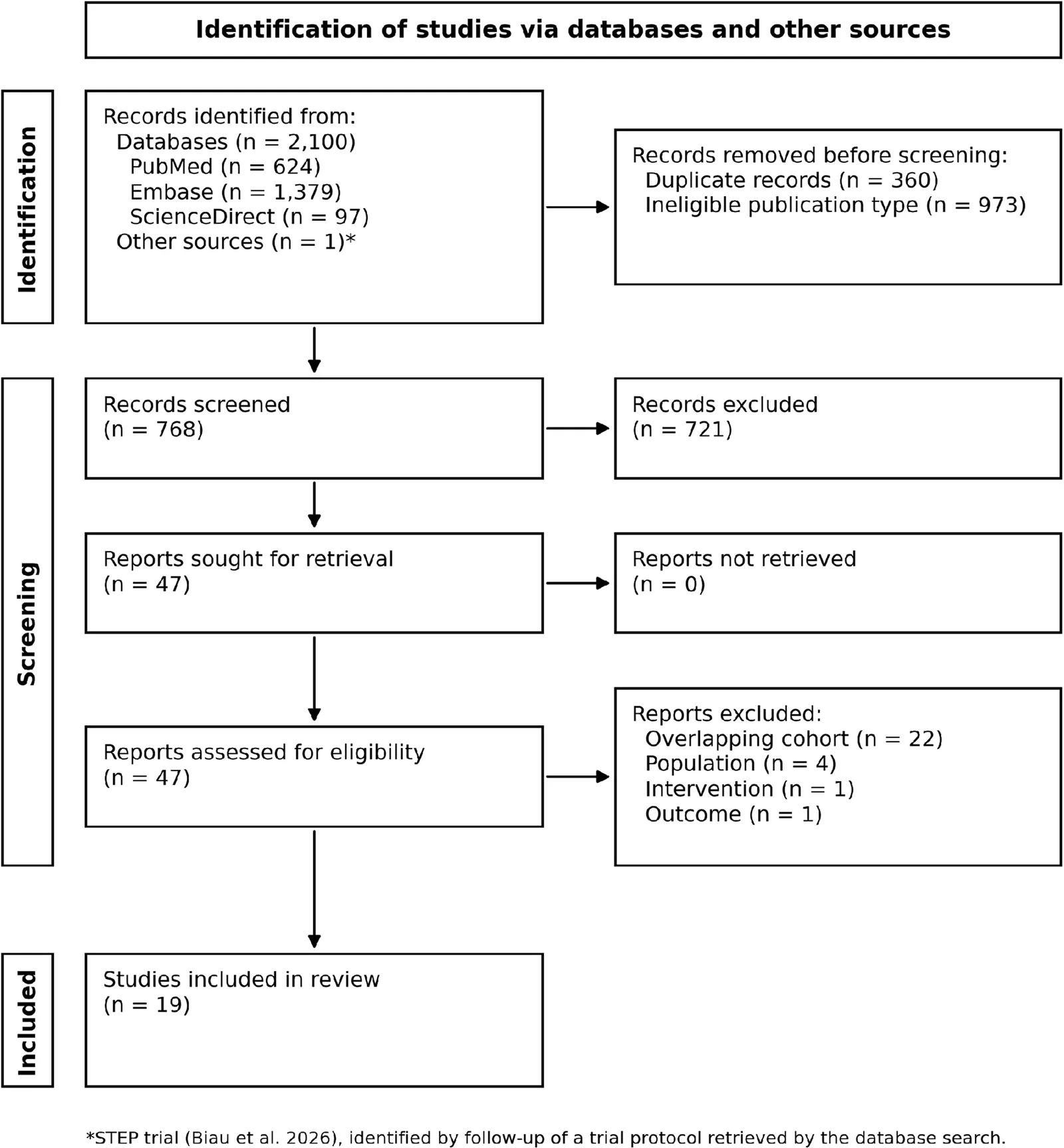
PRISMA 2020 flow diagram. Adapted from Page et al., BMJ 2021;372:n71 (CC BY 4.0)

The database search identified 2,100 records (PubMed 624, Embase 1,379, ScienceDirect 97), and one further record was identified by other methods. After removal of 360 duplicates and 973 records of an ineligible publication type, 768 records were screened and 721 were excluded. All 47 reports sought were retrieved and assessed in full text; 28 were excluded (22 overlapping cohorts, 4 population, 1 intervention, 1 outcome; Supplementary Table S6). Nineteen studies were included: 15 of pre-operative SRS and 4 of Cs-131 brachytherapy (Fig. 1).

### Study characteristics

The 19 studies comprised 1,125 patients and 1,157 BMs: 918 patients (940 BMs) treated with pre-operative SRS and 207 patients (217 BMs) treated with Cs-131 brachytherapy (Table 1). Pre-operative SRS studies were published from 2022 to 2026; 7 were prospective, 6 were retrospective, 1 combined a phase 1 trial with a retrospective expansion cohort, and 1 was a phase 2 trial [22]. All 4 brachytherapy studies (2017–2026) were prospective, including the Cs-131 arm of one randomized trial. Median age was 62 years with pre-operative SRS and 64 years with brachytherapy (study range 53–67 and 60–65 years). Baseline prognostic data were incompletely reported: KPS was reported in 4 of 15 pre-operative SRS studies and 3 of 4 brachytherapy studies (median 80 in each), extracranial disease status in 6 of 15 and 1 of 4, and concurrent systemic therapy in 6 of 15 and 2 of 4. Local recurrence was defined by RANO or RANO-BM criteria in 6 pre-operative SRS studies and 2 brachytherapy studies; the remaining studies used new or growing nodular enhancement, local investigator assessment, or independent central review (ROADS), and 2 pre-operative SRS studies did not report a definition.

**Table 1:** Characteristics of included studies

Study	Design	Patients (lesions)	Age, median	KPS, median	No extracranial​​disease, *n*/*N*	Concurrent systemic therapy, %	Most frequent primary	Local recurrence criteria	Median follow-up, mo
**Pre-operative SRS**									
Prabhu et al. (2023) [23] [PROPS-BM]	Retrospective (multi-institutional registries)^a^	404 (416)	60.6	NR	168/404	NR	Lung	Cavity LR by local investigator	18.1^b^
Li et al. (2022) [25]	Retrospective	24 (24)	64	NR	NR	NR	Lung	NR	16.5
Rogers et al. (2024) [26] [PREOP-1]	Prospective	12 (12)^c^	65	80	3/13	30.7	Lung	NR	6
Yoo et al. (2025) [27]	Retrospective	70 (70)	NR	NR	20/70	31	Melanoma	New nodular enhancement	NR
Marwaha et al. (2025) [38]	Prospective	31 (31)^d^	64	NR	7/31	NR	Lung	RANO	9
Hoyle et al. (2025) [37]	Phase 1 trial + retrospective expansion^e^	96 (96)	NR	NR	NR	NR	Lung	New nodular enhancement < 5 mm	9
Mallela et al. (2024) [39]	Prospective	26 (26)	NR^f^	80	NR	NR	Lung	RANO	≥ 12^g^
Kutuk et al. (2024) [40]	Retrospective	39 (47)^h^	62	80	21/47^i^	82.1^j^	Lung	RANO-BM (lesion-level)	14^k^
Deguchi et al. (2022) [41]	Retrospective	20 (20)	67	NR	NR	NR	Lung	New nodular enhancement	7.8^l^
Yaprak et al. (2025) [42]	Retrospective	18 (18)^m^	59	80	7/18	NR	Lung	RANO	15
Jansen et al. (2024) [43]	Prospective	21 (21)	NR^n^	NR	NR	NR	Melanoma	Contrast-enhancing mass within or adjacent to cavity	10.3
Agrawal et al. (2024) [44]	Prospective	47 (48)	59	NR	NR	25	Lung	RANO	14.7
Higazy et al. (2025) [45]	Prospective	35 (35)	61	NR	NR	48.6	Lung	Growing nodular enhancement	11.8
Schröder et al. (2025) [28]	Prospective	12 (13)^o^	53	NR	NR	31.3	Melanoma	Nodular enhancement in cavity (≥ 20% diameter increase)	13.8
Biau et al. (2026) [22] [STEP]	Prospective phase 2 trial^p^	63 (63)	62	NR	NR	NR	Lung	RANO	12.7
**Total / median**	**8 prospective**,** 6 retrospective**,** 1 mixed**	**918 (940)**	**62**	**80**	**—**	**—**	**Lung 12/15**	**—**	**—**
**Cs-131 brachytherapy**									
Weinberg et al. (2026) [20] [ROADS, Cs-131 arm]	Prospective RCT	103 (103)^q^	64	80	NR	48.5	Lung	Surgical-bed recurrence; independent central review	13.3
Kite et al. (2026) [46] [STaRT registry]	Prospective registry	51 (55)	64	80	NR	23.5	Lung	RANO	12.4
Wernicke et al. (2017) [31]	Prospective	32 (38)^r^	65	NR	NR	NR	Lung	New nodular/contrast enhancement ≤ 5 mm of cavity	11.9
Mahapatra et al. (2025) [47]	Prospective	21 (21)	60	80	4/21	NR	Lung	RANO-BM	2.93
**Total / median**	**4 prospective**	**207 (217)**	**64**	**80**	**—**	**—**	**Lung 4/4**	**—**	**—**

KPS, Karnofsky performance status; LR, local recurrence; NR, not reported; RANO(-BM), Response Assessment in Neuro-Oncology (Brain Metastases); RCT, randomized controlled trial

^a^ Retrospective and prospective institutional registries

^b^ Median imaging follow-up among surviving patients

^c^ 13 enrolled; 12 received pre-operative SRS and resection; outcomes in 6 evaluable patients

^d^ 20 on protocol and 11 treated off-study

^e^ Retrospective expansion of a phase 1 dose-escalation trial; denominator 96 as printed with the local-failure result (the cohort is given as 95 elsewhere in the paper)

^f^ Age reported only as a mean

^g^ Median not reported; all patients followed until death or ≥ 1 year after surgery

^h^ Source reports outcomes per lesion (47); patient-level counts (39) were analyzed

^i^ Lesion level; no extracranial disease in 21/47; oligometastatic (11) or widespread (15) extracranial disease

^j^ Any systemic therapy within 3 months, patient level (32/39)

^k^ Whole four-paradigm cohort

^l^ Mean

^m^ 18 resected index lesions (3 non-index lesions treated with SRS alone are not counted); 16 of 18 evaluable

^n^ Age reported only as means by arm

^o^ 12 patients with 13 resected metastases

^p^ Identified by other methods and published after the search date; KPS reported only in bands (90–100 in 64.2%)

^q^ One resection cavity per patient

^r^ 8 salvage lesions in previously irradiated sites excluded from all analyses; age, KPS, follow-up, tumor volume, and location are whole-cohort values (42 patients, 46 lesions) because the stratum values were not reported

### Treatment characteristics

Lung cancer was the most common primary in 12 of 15 pre-operative SRS studies and in all brachytherapy studies (Table 2). Pre-operative SRS used Gamma Knife in 9 studies, LINAC in 8, and CyberKnife in 4, with some studies using more than one platform. Among 12 studies reporting a numeric dose, the median prescription was 16.5 Gy (range 15–30). Three studies delivered fractionated treatment (Marwaha et al., 27 Gy in 3 fractions; Deguchi et al., 30–35 Gy in 5 fractions; STEP, 23.1 Gy in 3 fractions); two used mixed schedules (PROPS-BM, in which 78.8% of lesions were treated in a single fraction [16], and Jansen et al.); Kutuk et al. and Schröder et al. did not report a classifiable schedule; and the remaining 8 studies used single-fraction SRS. Median treated volume was 12.2 cm³ (range 9.6–17.6) for pre-operative SRS and 12.1 cm³ (range 10.7–13.5) for brachytherapy. Two brachytherapy studies used collagen-embedded tiles prescribed to 60 Gy at 5 mm (Weinberg, Kite), and two used stranded seeds prescribed to 80 Gy at 5 mm (Wernicke, Mahapatra).

**Table 2:** Treatment characteristics

Study	Platform	Volume, cm³ (median)	Supratentorial, *n*/*N*	Prescription dose	Fractions	Fractionation group (analysis)	Isodose line, %	Margin
Pre-operative SRS								
Prabhu et al. (2023)	LINAC, GK, CK	10.0	311/416	15 Gy (SF); 24 Gy (MF)	1 (78.8% of lesions); 3–5	Mixed^a^	NR	0 mm in 53.6%
Li et al. (2022)	GK	10.1	NR	17 Gy	1	Single	50	None
Rogers et al. (2024)	LINAC	9.6	10/13	16 Gy	1	Single	70.4	Yes
Yoo et al. (2025)	GK	NR	NR	15–20 Gy	1	Single	50	NR
Marwaha et al. (2025)	GK Icon	12.5	23/31	27 Gy (15–27)	3 (1–3)	Fractionated^b^	50	None
Hoyle et al. (2025)	LINAC	NR	56/95	15 Gy	1	Single	NR	None
Mallela et al. (2024)	CK, GK, LINAC	12.2	24/26	17.3 Gy	1	Single	80	Yes
Kutuk et al. (2024)	GK Icon	NR	37/47	NR^c^	1	Not classified	≥ 50	None
Deguchi et al. (2022)	LINAC	17.6	20/20	30–35 Gy^d^	5	Fractionated	NR	Yes
Yaprak et al. (2025)	CK	14.19	5/18	15 Gy (12–17)	1 (1–2)	Single	84	Yes
Jansen et al. (2024)	NR	NR	NR	16 Gy (SF); 24–27 Gy (MF)^e^	1 or 3	Mixed	NR	NR
Agrawal et al. (2024)	GK, LINAC	NR	34/48	15 Gy	1	Single	50	None
Higazy et al. (2025)	GK, CK	15.0	30/35	17 Gy	1	Single	NR	Yes
Schröder et al. (2025)	GK, LINAC	10.4	12/16	NR	3, 5, or other	Not classified^f^	80–90	GK 0 mm; LINAC 1 mm
Biau et al. (2026) [STEP]	LINAC	NR	53/63	23.1 Gy (3 × 7.7 Gy)	3	Fractionated	70	Yes
**Median (range)**	**—**	**12.2 (9.6–17.6)**	**—**	**16.5 Gy (15–30)**	**1 (1–5)**	**8 single / 3 fractionated / 2 mixed / 2 not classified**	**—**	**—**
**Cs-131 brachytherapy**								
Weinberg et al. (2026)	Collagen-embedded tiles (GammaTile)	NR	83/103	60 Gy at 5 mm	Permanent implant	60 Gy tile	N/A	N/A
Kite et al. (2026)	Collagen-embedded tiles (GammaTile)	NR	48/55	60 Gy at 5 mm	Permanent implant	60 Gy tile	N/A	N/A
Wernicke et al. (2017)	Stranded seeds	13.45	35/46	80 Gy at 5 mm	Permanent implant	80 Gy seeds	N/A	N/A
Mahapatra et al. (2025)	Stranded seeds	10.65	13/21	80 Gy at 5 mm	Permanent implant	80 Gy seeds	N/A	N/A
**Median (range)**	**—**	**12.1 (10.7–13.5)**	**—**	**60–80 Gy at 5 mm**	**—**	**2 tile / 2 seed**	**—**	**—**

CK, CyberKnife; GK, Gamma Knife; LINAC, linear accelerator; MF, multifraction; N/A, not applicable; NR, not reported; SF, single fraction. ^a^ Single fraction in 328/416 lesions (78.8%); 12-month outcomes not reported by fractionation, so classified as mixed (counted as single-fraction in a sensitivity analysis). ^b^ 11 off-study patients received 1–3 fractions. ^c^ Dose not reported in the main text; single-session pre-operative SRS delivered 1–7 days before resection, with 3–5 fraction SRS a separate treatment paradigm. ^d^ 30 Gy (*n* = 11) or 35 Gy (*n* = 9); median 30 Gy used for the summary. ^e^ 16 Gy in 1 ^f^ raction (15 patients) or 24–27 Gy in 3 fractions (6 patients). f Schedules other than 3 or 5 fractions not described for 5 of 16 tumors

### Risk of bias

MINORS scores ranged from 6 to 15 (Supplementary Table S2). Two studies (ROADS, STEP) were at low risk of bias, 15 were at moderate risk, and 2 (Li, Yoo) were at high risk. The most common deficiencies were retrospective data collection, the absence of blinded or central outcome assessment in all studies except ROADS and STEP, and the absence of a prospective sample-size calculation.

### Local control

**Fig. 2:**
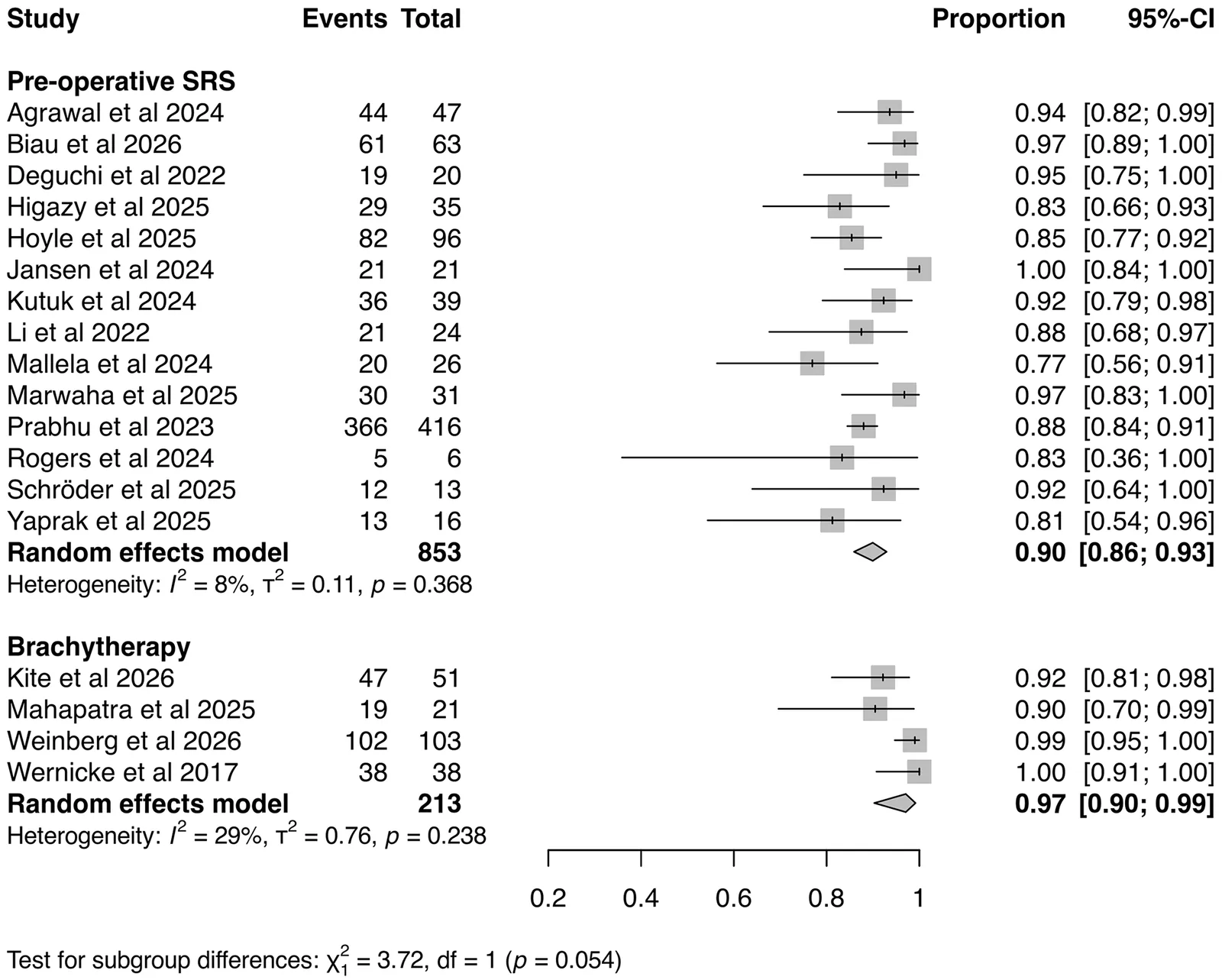
Twelve-month local control after pre-operative SRS and Cs-131 brachytherapy. Squares show each study’s proportion with exact (Clopper–Pearson) 95% confidence intervals; diamonds show pooled proportions for each modality from random-effects generalized linear mixed models. The test for subgroup differences uses separate between-study variances. Counts were observed for Hoyle, Kutuk, Marwaha, Rogers, Schröder, Weinberg, and Wernicke and were reconstructed by multiplying a published 12-month Kaplan–Meier or rate estimate by the analyzed sample size for the remaining studies (Supplementary Table S4). Wernicke et al.: treatment-naïve lesions only (38 of 46). Yoo did not report 12-month local control; Hoyle contributed a crude count over follow-up. Rogers and Yaprak contributed 6 and 16 evaluable patients, respectively

Pooled 12-month LC was 90% (95% CI 86–93; 14 studies; τ² = 0.11, I² = 8%) after pre-operative SRS and 97% (95% CI 90–99; 4 studies; τ² = 0.76, I² = 29%) after Cs-131 brachytherapy (test for subgroup differences χ²₁ = 3.72, *p* = 0.054; Fig. 2; Table 3). Meta-regression gave β = 1.16 (95% CI 0.20–2.13; *p* = 0.019), and the Hartung–Knapp model gave *p* = 0.066. Yoo et al. did not report 12-month LC.

The difference was not statistically significant after excluding ROADS (Cs-131 95%; *p* = 0.209), PROPS-BM (*p* = 0.077), or Wernicke et al. (*p* = 0.120), with inverse-variance Hartung–Knapp pooling (*p* = 0.066), or when the analysis was restricted to studies with median follow-up of at least 12 months (*p* = 0.101) or to patient-level denominators (*p* = 0.168) (Supplementary Table S3). It reached significance when any one of six studies was excluded (STEP, Jansen et al., Marwaha et al., Kite et al., Agrawal et al., and Deguchi et al.; *p* = 0.025–0.048), including the analysis without STEP (*p* = 0.025); when the analysis was restricted to studies with observed counts (90% vs. 99%; *p* = 0.009); and when crude counts replaced rate-derived counts (*p* = 0.001). Within pre-operative SRS, LC after hypofractionated SRS (96%, 95% CI 91–99; 3 studies, 110/114) was higher than after single-fraction SRS (86%, 95% CI 81–89; 7 studies, 214/250; *p* = 0.005), and the difference persisted when mixed-schedule studies were counted as single-fraction (87%; *p* = 0.009).

### Leptomeningeal disease and radiation necrosis

**Fig. 3:**
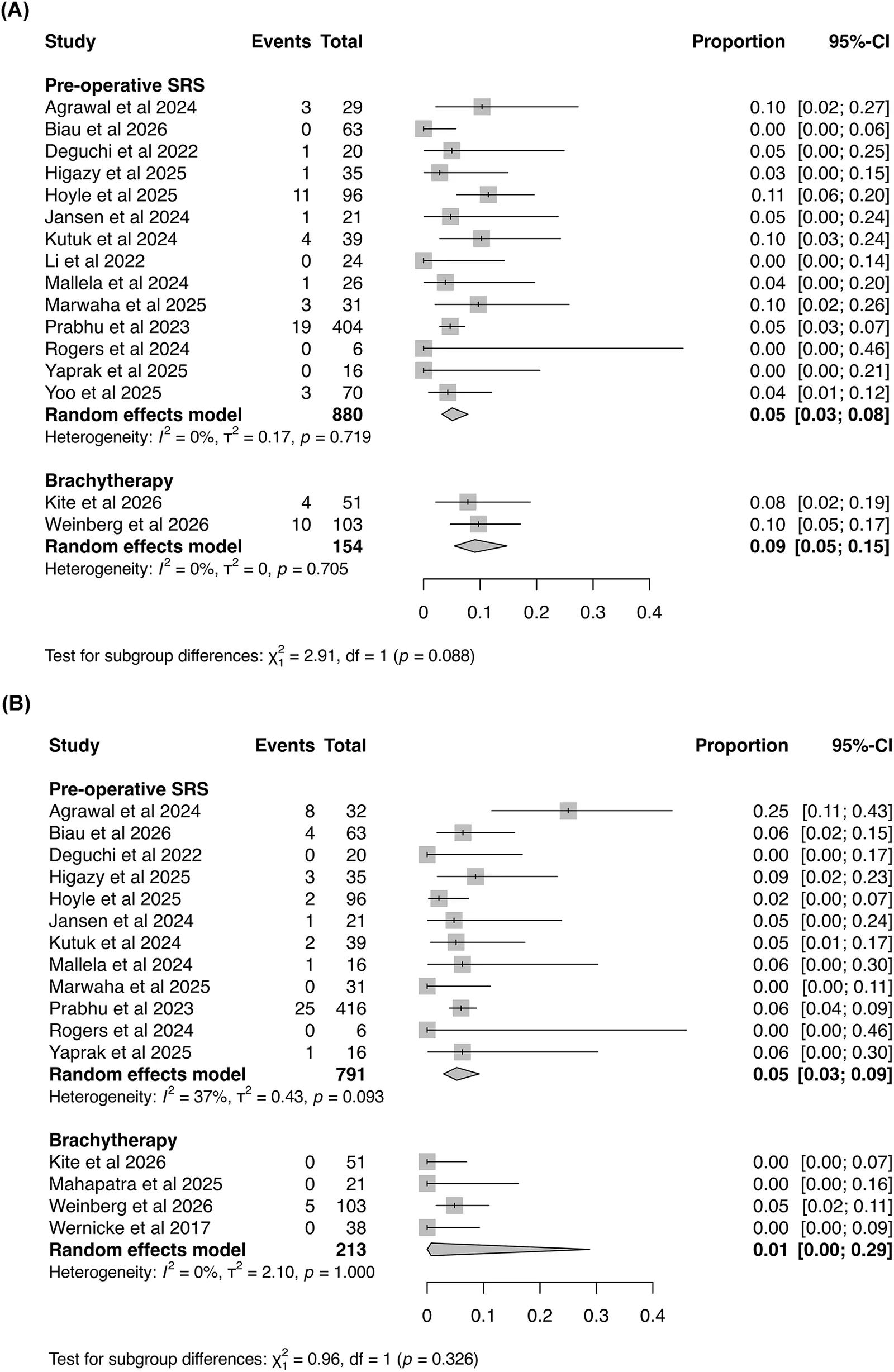
(**A**) Leptomeningeal disease and (**B**) radiation necrosis after pre-operative SRS and Cs-131 brachytherapy. Conventions as in Fig. 2. Kite et al.: zero RN events confirmed from registry data. Schröder, Mahapatra, and Wernicke did not report LMD; Li, Schröder, and Yoo did not report RN. Agrawal et al.: LMD and RN counted among patients with follow-up data (Supplementary Table S4). Mallela et al. reported RN for the 16 patients evaluable at 12 months

LMD occurred in 5.1% (95% CI 3.3–7.8; 14 studies) after pre-operative SRS and 9.1% (95% CI 5.5–15; 2 studies) after Cs-131 brachytherapy (*p* = 0.088; Fig. 3A); with inverse-variance Hartung–Knapp pooling, *p* = 0.256. RN occurred in 5.2% (95% CI 2.9–9.2; 12 studies) and 0.7% (95% CI < 0.1–29; 4 studies), respectively (*p* = 0.326; Fig. 3B). All 5 brachytherapy RN events occurred in ROADS; the other three Cs-131 studies reported none, so an analysis of RN without ROADS was not estimable. Schröder et al., Mahapatra et al., and Wernicke et al. did not report LMD, and Li et al., Schröder et al., and Yoo et al. did not report RN. Because few brachytherapy studies and events contributed, both comparisons are underpowered and are interpreted descriptively.

### Overall survival

Median OS, summarized as the median of study-level medians, was 14.4 months (range 6.3–26.4; 10 studies) with pre-operative SRS and 15.6 months (range 5.9–42.5; 4 studies) with brachytherapy. The upper limit of the brachytherapy range reflects the ROADS modified intention-to-treat median; excluding it, the range was 5.9–20.1 months.

**Table 3:** Pooled outcomes by modality

Outcome	k (SRS)	Pre-op SRS, % (95% CI)	τ² / I²	k (Cs-131)	Cs-131, % (95% CI)	τ² / I²	Subgroup test χ² (*p*), primary	Meta-regression β (95% CI), *p*	Hartung–Knapp *p*
12-month local control	14	90 (86–93)	0.11 / 8%	4	97 (90–99)	0.76 / 29%	3.72 (0.054)	1.16 (0.20 to 2.13), 0.019	0.066
Leptomeningeal disease	14	5.1 (3.3–7.8)	0.17 / 0%	2	9.1 (5.5–15)	0.00 / 0%	2.91 (0.088)	0.55 (− 0.19 to 1.29), 0.144	0.256
Radiation necrosis	12	5.2 (2.9–9.2)	0.43 / 37%	4	0.7 (< 0.1–29)	2.10 / 0%	0.96 (0.326)	−1.27 (− 2.71 to 0.17), 0.084	0.191
Median overall survival, mo (descriptive)	10	14.4 (range 6.3–26.4)	—	4	15.6 (range 5.9–42.5; 5.9–20.1 excluding ROADS)	—	Not tested	—	—

Pooled estimates from random-effects generalized linear mixed models (logit link, maximum likelihood), fitted separately for each modality. Primary test: test for subgroup differences with separate between-study variances. Secondary: mixed-effects meta-regression with modality as moderator (shared τ²) and inverse-variance pooling with Hartung–Knapp adjustment. I² from the Wald-type test of heterogeneity in the mixed model, as shown in Figs. 2 and 3. Median OS is the median of study-level medians; the ROADS value (42.5 months) is the modified intention-to-treat median

## Discussion

### Summary of findings

In this meta-analysis of 19 studies, pre-operative SRS and Cs-131 brachytherapy were both associated with high 12-month LC and low rates of LMD and RN after resection of treatment-naïve BMs. Notably, the wider between-study variance in the brachytherapy subgroup ($\:{\tau\:}^{2}=0.76$ vs. * 0.11*), which rendered the primary subgroup test and Hartung–Knapp model borderline, may partly reflect the reconstruction of 12-month event counts from Kaplan–Meier estimates in cohorts with censoring prior to 1 year, which assigns full-sample weight to tail estimates and amplifies between-study dispersion on the logit scale. Consistent with this, restricting the analysis to observed counts (* p=0.009*) or substituting crude counts (* p=0.001*) yielded a statistically significant difference; in leave-one-out sensitivity analyses (* p=0.025-0.209*), LC favored brachytherapy with every exclusion, although statistical significance depended on which studies were included. Because this comparison is between single-arm cohorts that differ in patient selection, dose, outcome definition, and follow-up, it is exploratory. Within pre-operative SRS, hypofractionated treatment achieved LC of 96%, similar to brachytherapy and higher than single-fraction treatment (86%; *p* = 0.005). LMD and RN did not differ detectably, and median OS was similar.

### Local control

Post-operative SRS achieves 12-month cavity control in approximately 80%–90% of patients, but irregular cavity morphology, uncertain coverage of the surgical tract, and cavity remodeling complicate treatment [48–50]. Consensus guidelines recommend expanding the post-operative clinical target volume (CTV) along the dura and surgical tract [9, 10], which increases the volume of irradiated normal brain [51]. In a randomized comparison, Yeboa et al. reported less incomplete treatment with pre-operative than with post-operative SRS (12% vs. 27%) [52]. Pre-operative SRS treats the intact metastasis without a cavity CTV expansion and improves conformity and gradient indices [53, 54]. Our pooled pre-operative estimate of 90% is consistent with earlier pre-operative series and with a meta-analysis of comparative studies that found similar local failure after pre-operative and post-operative SRS [15, 30, 55].

Dose and fractionation may explain much of the observed difference. The pooled pre-operative SRS estimate was dominated by single-fraction treatment, usually 15–17 Gy, delivered to targets with a median volume of 12 cm³. Local failure after pre-operative SRS clusters in tumors larger than 3 cm or 8–10 cm³ [15, 25], and in PROPS-BM, single-fraction SRS was associated with higher 2-year cavity recurrence than 3–5 fraction SRS (16.3% vs. 2.9%) [16]. Consistent with these data, the three hypofractionated pre-operative SRS studies achieved LC of 96% (110/114 patients), higher than single-fraction pre-operative SRS (86%; *p* = 0.005) and similar to brachytherapy. Although this subgroup comprised three studies, it suggests that the apparent advantage of brachytherapy may partly reflect dose de-escalation in single-fraction pre-operative SRS rather than an intrinsic property of either modality. Fractionated pre-operative regimens are increasingly used [38, 56], and ongoing randomized trials comparing pre-operative and post-operative SRS (NCT03750227, NCT05438212) will further define the role of pre-operative treatment.

Cs-131 brachytherapy begins radiation at the time of resection and removes the interval between surgery and adjuvant treatment. Earlier iodine-125 series reported inconsistent outcomes and substantial toxicity, whereas Cs-131 series have reported 12-month LC of 92%–100% [17, 19, 20, 31, 46]. The pooled brachytherapy estimate, however, rests on four studies that differ in implant (collagen tiles vs. stranded seeds), prescription (60 vs. 80 Gy at 5 mm), recurrence definition, and follow-up, and ROADS contributed nearly half of the brachytherapy patients.

Both strategies require multidisciplinary coordination. Pre-operative SRS requires radiosurgery planning and delivery before surgery and is generally unsuitable when urgent decompression is needed or the diagnosis is uncertain [14]. Brachytherapy requires advance ordering of radioactive sources, intraoperative participation of radiation oncology and medical physics, and institutional licensing, which may also limit its use in urgent cases [18, 19]. Costs also differ: pre-operative SRS uses existing radiosurgery infrastructure and replaces the post-operative course, whereas brachytherapy adds the cost of the implant but avoids a separate course of external-beam planning and delivery. No published analysis has compared the costs of pre-operative SRS and Cs-131 brachytherapy. In a single-institution analysis of 49 patients, resection with Cs-131 seed brachytherapy had lower direct hospital costs than resection with post-operative SRS ($19,271 vs. $44,219 per patient) and was more cost-effective [57]; however, that analysis was based on hospital charges, predates collagen-embedded tiles, and did not include pre-operative SRS. Pre-operative SRS has not been costed, but it substitutes for rather than adds to the post-operative course. Formal comparisons should account for local recurrence, salvage treatment, and toxicity. The two approaches may therefore serve complementary roles, with selection guided by clinical urgency, tumor size and location, diagnostic certainty, and institutional resources. For example, patients with a solitary brain metastasis may benefit from brachytherapy, as it can facilitate timely surgical intervention and potentially reduce hospital length of stay. In contrast, patients with multiple brain metastases may benefit from pre-operative SRS directed both to the index lesion planned for resection and to unresected lesions, thereby shortening the time to comprehensive CNS disease control.

### Radiation necrosis and leptomeningeal disease

RN rates were low with both approaches. Pre-operative SRS treats a smaller, well-defined target and reduces the volume of normal brain receiving 10–14 Gy compared with post-operative plans [53]. Collagen-embedded Cs-131 provides consistent source spacing, and its short half-life (9.7 days) delivers most of the dose before substantial cavity involution [19]. However, the pooled brachytherapy RN estimate of 0.7% rests on 5 events, all from ROADS, and its confidence interval extends to 29%, so it does not exclude a rate similar to that of pre-operative SRS. In ROADS, RN was similar after Cs-131 brachytherapy and post-operative stereotactic radiotherapy (5.3% vs. 5.7%) [20].

LMD was numerically more frequent after brachytherapy than after pre-operative SRS and only two brachytherapy studies reported LMD. In ROADS, LMD was likewise numerically higher with brachytherapy than with post-operative stereotactic radiotherapy (9.7% vs. 3.0%; *p* = 0.15) [20]. Pre-operative SRS may reduce dissemination by devitalizing tumor cells before surgical manipulation [15, 37], whereas intracavitary sources irradiate the cavity surface but not cells already released into the CSF. Breast histology, posterior fossa location, and piecemeal resection influence LMD risk, and few studies distinguished classical from nodular LMD, which differ in prognosis [11, 41, 58, 59]. Both approaches demonstrated lower rates of LMD than the median incidence of approximately 16% reported after surgical resection of brain metastases in a prior meta-analysis [11]. LMD was numerically higher with brachytherapy than with pre-operative SRS (9.1% vs. 5.1%), although this difference was not statistically significant (*p* = 0.088).

### Overall survival

Median OS was similar between groups. Survival after resection of BMs is largely determined by extracranial disease control and systemic therapy [60], which were incompletely reported, so differences in intracranial control would not be expected to translate into survival differences in this analysis. The ROADS median OS of 42.5 months exceeds that of the other studies and was reported for the modified intention-to-treat population [20].

### Limitations

This analysis has several limitations. First, it compares single-arm cohorts across studies rather than patients within studies and cannot account for differences in patient selection, tumor size, histology, systemic therapy, or surveillance. Second, the definition of local failure differed between studies and between modalities (Table 1), ranging from RANO or RANO-BM to new nodular enhancement, local investigator assessment, and independent central review; because LC is the primary endpoint, this heterogeneity directly limits comparability. Third, 11 of 18 LC counts were reconstructed from published 12-month Kaplan–Meier or cumulative-incidence estimates, which treats all patients as followed to 12 months and does not account for the competing risk of death; in the four-study brachytherapy subgroup, applying full-cohort denominators to censored actuarial estimates inflated between-study heterogeneity ($\:{\tau\:}^{2}=0.76$) and widened the Hartung–Knapp confidence interval (* p=0.066*), whereas analyses using observed counts (* p=0.009*), crude counts (* p=0.001*), or 6 of 18 leave-one-out exclusions (* p=0.025-0.048*) reached statistical significance. Counts were lesion-level where reported (PROPS-BM, Schröder, Wernicke) and patient-level in studies that reported only patients despite multiple lesions (Agrawal, Kutuk, Kite); in the remaining studies each patient had one lesion. Fourth, the brachytherapy estimate rests on four studies that differed in implant and prescription, one of which (ROADS) contributed nearly half of the patients, was industry-sponsored, and was co-authored by a study author; the LC difference was not significant on the primary test and remained non-significant without ROADS, PROPS-BM, or Wernicke et al., with inverse-variance pooling, or when restricted to studies with at least 12 months of follow-up.

Fifth, concurrent systemic therapy, including CNS-active targeted therapy and immunotherapy, was reported in only 6 of 15 pre-operative SRS and 2 of 4 brachytherapy studies, and extracranial disease status in 6 of 15 and 1 of 4; these factors may influence recurrence, LMD, RN, and survival. Sixth, units of analysis were mixed (lesion-level where reported, patient-level otherwise), although cohorts averaged 1.03 lesions per patient and an analysis restricted to patient-level denominators gave a similar estimate (91% vs. 96%; *p* = 0.168). Seventh, most studies were at moderate risk of bias, six pre-operative SRS studies were retrospective, and several studies had median follow-up shorter than the 12-month endpoint. Finally, the protocol was not registered in PROSPERO, several sensitivity analyses were added during peer review, and publication bias could not be assessed.

### Future directions

Although both approaches achieve high LC, ROADS showed that a difference in surgical bed control can be detected against post-operative SRS despite its high LC in prior trials [5]. A prospective head-to-head comparison of pre-operative SRS and Cs-131 brachytherapy would add value in resolving this question. Prospective registries and comparative cohorts with standardized recurrence definitions, lesion-level reporting, competing-risk analyses, and systemic therapy data would complement such a trial. Comparisons with fractionated pre-operative SRS, analyses of the patient and tumor factors that favor either approach, dosimetric predictors of RN, and cost-effectiveness analyses are also needed.

## Conclusions

Pre-operative SRS and Cs-131 brachytherapy were both associated with high 12-month LC and low rates of LMD and RN after resection of treatment-naïve BMs. LC trended higher with Cs-131 brachytherapy than with pre-operative SRS overall (97% vs. 90%; *p* = 0.054) and significantly higher among studies reporting observed counts (99% vs. 90%; *p* = 0.009), whereas hypofractionated pre-operative SRS achieved LC similar to that of brachytherapy (96% vs. 97%), suggesting that the difference may partly reflect single-fraction dose de-escalation. The two approaches may serve complementary roles, with selection guided by clinical urgency, tumor size and location, diagnostic certainty, and institutional resources.

## Supplementary Information

Below is the link to the electronic supplementary material.

Supplementary Material 1: Supplementary Table S1. Search strategy for each database. Supplementary Table S2. MINORS risk-of-bias assessment. Supplementary Table S3. Sensitivity analyses. Supplementary Table S4. Study-level outcome data and derivation of 12-month counts. Supplementary Table S5. PRISMA 2020 checklist. Supplementary Table S6. Reports assessed in full text and excluded, with reasons. Supplementary Figure S1. Twelve-month local control, inverse-variance pooling (REML, Hartung–Knapp). Supplementary Figure S2. Leptomeningeal disease, inverse-variance pooling (REML, Hartung–Knapp). Supplementary Figure S3. Radiation necrosis, inverse-variance pooling (REML, Hartung–Knapp).

## Data Availability

All data supporting the findings of this study are available within the paper and its Supplementary Information.

## Code availability

The R script that produced all pooled estimates, tables, and figures is available upon reasonable request.

## Declarations

### Use of artificial intelligence

A large language model (Claude, Anthropic) was used to locate and cross-check extracted values against source pages, and to assist with editing the text; a code assistant (Codex, OpenAI) was used to help write the R analysis script. All screening decisions, extracted values, analyses, and text were reviewed and verified by the authors, who take full responsibility for the content.
