# Supplementary material for: Pre-operative stereotactic radiosurgery versus intraoperative cesium-131 brachytherapy for resected brain metastases: a systematic review and meta-analysis
Source: J Neurooncol. 2026 Nov 9;180(2):40. doi: 10.1007/s11060-026-05870-3 (PMC13748270; doi:10.1007/s11060-026-05870-3)
Supplement: Supplementary file 1 [file 11060_2026_5870_MOESM1_ESM.docx]

**Supplementary Material**

*Pre-Operative Stereotactic Radiosurgery Versus Intraoperative Cesium-131 Brachytherapy for Resected Brain Metastases: A Systematic Review and Meta-Analysis. Journal of Neuro-Oncology.*

**Supplementary Table S1. Search strategy for each database**

| **Database** | **Cohort** | **Date run** | **Search string** | **Fields / filters** | **Records exported** | **Records after removal of ineligible publication types** |
| --- | --- | --- | --- | --- | --- | --- |
| PubMed/MEDLINE | Pre-operative SRS | 1 Oct 2026 | ("Radiosurgery"[Mesh] OR radiosurgery[tiab] OR SRS[tiab] OR "stereotactic radiosurgery"[tiab] OR SRT[tiab] OR "stereotactic radiotherapy"[tiab] OR "gamma knife"[tiab] OR gammaknife[tiab] OR cyberknife[tiab] OR "linear accelerator"[tiab] OR LINAC[tiab]) AND (preoperative[tiab] OR pre-operative[tiab] OR "pre operative"[tiab] OR neoadjuvant[tiab] OR neo-adjuvant[tiab] OR "pre-resection"[tiab] OR "prior to resection"[tiab] OR "prior to surgery"[tiab] OR "before resection"[tiab] OR "before surgery"[tiab] OR presurgical[tiab] OR "pre-surgical"[tiab]) AND ("Brain Neoplasms/secondary"[Mesh] OR "brain metastas*"[tiab] OR "cerebral metastas*"[tiab] OR "intracranial metastas*"[tiab] OR "brain metastatic"[tiab] OR "metastatic brain"[tiab] OR "brain tumor*"[tiab] OR "brain tumour*"[tiab]) | None | 286 | 187 |
| PubMed/MEDLINE | Cs-131 brachytherapy | 1 Oct 2026 | ("Brachytherapy"[Mesh] OR brachytherapy[tiab] OR "Cesium Radioisotopes"[Mesh] OR "cesium-131"[tiab] OR "caesium-131"[tiab] OR "Cs-131"[tiab] OR "Cs131"[tiab] OR "131Cs"[tiab] OR GammaTile[tiab] OR "Gamma Tile"[tiab] OR "intracavitary radiation"[tiab] OR "intraoperative radiotherapy"[tiab] OR "radioactive seed*"[tiab] OR "permanent implant*"[tiab]) AND ("Brain Neoplasms/secondary"[Mesh] OR "brain metastas*"[tiab] OR "cerebral metastas*"[tiab] OR "intracranial metastas*"[tiab] OR "metastatic brain"[tiab] OR "resection cavity"[tiab] OR "surgical cavity"[tiab] OR "resected brain"[tiab]) | None | 338 | 198 |
| Embase | Pre-operative SRS | 1 Oct 2026 | ('radiosurgery'/exp OR 'stereotactic radiosurgery'/exp OR radiosurgery:ti,ab OR SRS:ti,ab OR 'stereotactic radiotherapy':ti,ab OR SRT:ti,ab OR 'gamma knife':ti,ab OR cyberknife:ti,ab OR LINAC:ti,ab OR 'linear accelerator':ti,ab) AND (preoperative:ti,ab OR 'pre-operative':ti,ab OR neoadjuvant:ti,ab OR 'neo-adjuvant':ti,ab OR 'pre-resection':ti,ab OR 'before surgery':ti,ab OR 'before resection':ti,ab OR presurgical:ti,ab OR 'pre-surgical':ti,ab) AND ('brain metastasis'/exp OR 'brain metastas*':ti,ab OR 'cerebral metastas*':ti,ab OR 'intracranial metastas*':ti,ab OR 'metastatic brain':ti,ab) | None | 501 | 224 |
| Embase | Cs-131 brachytherapy | 1 Oct 2026 | ('brachytherapy'/exp OR brachytherapy:ti,ab OR 'cesium 131'/exp OR 'cesium-131':ti,ab OR 'caesium-131':ti,ab OR 'Cs-131':ti,ab OR '131Cs':ti,ab OR GammaTile:ti,ab OR 'intracavitary radiation':ti,ab OR 'intraoperative radiotherapy':ti,ab OR 'radioactive seed*':ti,ab) AND ('brain metastasis'/exp OR 'brain metastas*':ti,ab OR 'cerebral metastas*':ti,ab OR 'intracranial metastas*':ti,ab OR 'resection cavity':ti,ab OR 'resected brain':ti,ab) | None | 878 | 421 |
| ScienceDirect | Pre-operative SRS (pass 1a) | 30 Oct 2026 | ("stereotactic radiosurgery" OR "stereotactic radiotherapy") AND (preoperative OR neoadjuvant OR "pre-operative") AND ("brain metastases" OR "brain metastasis" OR "cerebral metastases") | Title, abstract, keywords; research articles | 37 |  |
| ScienceDirect | Pre-operative SRS (pass 1b) | 30 Oct 2026 | ("gamma knife" OR cyberknife) AND (preoperative OR neoadjuvant OR "pre-operative") AND ("brain metastases" OR "brain metastasis" OR "cerebral metastases") | Title, abstract, keywords; research articles | 6 |  |
| ScienceDirect | Pre-operative SRS (pass 2) | 30 Oct 2026 | (SRS OR LINAC OR radiosurgery) AND (preoperative OR neoadjuvant OR "pre-operative") AND ("brain metastases" OR "brain metastasis" OR "cerebral metastases") | Title, abstract, keywords; research articles | 40 | 43 (3 passes combined) |
| ScienceDirect | Cs-131 brachytherapy | 30 Oct 2026 | (brachytherapy OR "cesium-131" OR "Cs-131" OR GammaTile) AND ("brain metastases" OR "brain metastasis" OR "cerebral metastases") | Title, abstract, keywords; research articles | 14 | 14 |
| Other methods | Pre-operative SRS | 30 Oct 2026 | STEP trial (Biau et al. 2026), identified by following up a trial protocol retrieved by the database search | — | 1 | 1 |

PubMed/MEDLINE and Embase were searched from inception to 1 October 2026; ScienceDirect was searched on 30 October 2026. Reference lists of included studies and relevant reviews were hand-searched.

Records exported: 2,100. Before import to Rayyan, 973 records of database-assigned publication types excluded by the eligibility criteria (conference abstracts, reviews and meta-analyses, case reports, letters, editorials, notes, comments, errata) and 40 records overlapping between ScienceDirect passes were removed. The remaining 1,087 records were imported to Rayyan, 320 further duplicates were removed, and 767 database records plus 1 record identified by other methods (768) were screened.

ScienceDirect limits each field to 8 Boolean connectors and does not support truncation, so the pre-operative strategy was run as three passes whose union is the full strategy. No language filter was applied in the database searches.

**Supplementary Table S2. Methodological quality of included studies assessed with the Methodological Index for Non-Randomized Studies (MINORS), non-comparative items**

| **Study** | **Design** | **1** | **2** | **3** | **4** | **5** | **6** | **7** | **8** | **Total (/16)** | **Risk of bias** |
| --- | --- | --- | --- | --- | --- | --- | --- | --- | --- | --- | --- |
| **Pre-operative SRS** | | | | | | | | | | | |
| Prabhu 2023 (PROPS-BM) | Retrospective/prospective registries | 2 | 1 | 1 | 2 | 1 | 2 | 1 | 0 | **10** | Moderate |
| Li 2022 | Retrospective | 2 | 1 | 0 | 2 | 0 | 2 | 1 | 0 | **8** | High |
| Rogers 2024 (PREOP-1) | Prospective feasibility trial | 2 | 1 | 2 | 2 | 0 | 1 | 0 | 1 | **9** | Moderate |
| Yoo 2025 | Retrospective | 2 | 1 | 0 | 2 | 1 | 0 | 0 | 0 | **6** | High |
| Marwaha 2025 | Prospective pilot + off-study | 2 | 1 | 1 | 2 | 1 | 1 | 1 | 1 | **10** | Moderate |
| Hoyle 2025 | Phase 1 trial + retrospective expansion | 2 | 1 | 1 | 2 | 1 | 1 | 1 | 1 | **10** | Moderate |
| Mallela 2024 | Prospective | 2 | 1 | 2 | 2 | 1 | 2 | 1 | 0 | **11** | Moderate |
| Kutuk 2024 | Retrospective | 2 | 2 | 0 | 2 | 1 | 2 | 1 | 0 | **10** | Moderate |
| Deguchi 2022 | Retrospective | 2 | 2 | 0 | 2 | 1 | 1 | 1 | 0 | **9** | Moderate |
| Yaprak 2025 | Retrospective | 2 | 1 | 0 | 2 | 1 | 2 | 1 | 0 | **9** | Moderate |
| Jansen 2024 | Prospective | 2 | 1 | 2 | 2 | 1 | 1 | 0 | 2 | **11** | Moderate |
| Agrawal 2024 | Prospective | 2 | 1 | 2 | 2 | 1 | 2 | 0 | 2 | **12** | Moderate |
| Higazy 2025 | Prospective | 2 | 1 | 2 | 2 | 1 | 1 | 1 | 2 | **12** | Moderate |
| Schröder 2025 | Prospective | 2 | 1 | 2 | 2 | 1 | 2 | 1 | 1 | **12** | Moderate |
| Biau 2026 (STEP) | Prospective phase 2 trial | 2 | 1 | 2 | 2 | 2 | 2 | 2 | 2 | **15** | Low |
| **Cs-131 brachytherapy** | | | | | | | | | | | |
| Weinberg 2026 (ROADS, Cs-131 arm)ᵃ | Randomized controlled trial | 2 | 1 | 2 | 2 | 2 | 2 | 2 | 2 | **15** | Low |
| Kite 2026 (STaRT registry) | Prospective registry | 2 | 1 | 2 | 2 | 1 | 2 | 0 | 0 | **10** | Moderate |
| Wernicke 2017 | Prospective | 2 | 1 | 2 | 2 | 2 | 1 | 1 | 1 | **12** | Moderate |
| Mahapatra 2025 | Prospective | 2 | 1 | 2 | 2 | 1 | 1 | 1 | 0 | **10** | Moderate |

Items: 1, clearly stated aim; 2, inclusion of consecutive patients; 3, prospective collection of data; 4, endpoints appropriate to the aim of the study; 5, unbiased assessment of the study endpoint; 6, follow-up period appropriate to the aim of the study; 7, loss to follow-up <5%; 8, prospective calculation of the study size. Each item was scored 0 (not reported), 1 (reported but inadequate), or 2 (reported and adequate); maximum 16. Risk of bias: ≥13 low, 9–12 moderate, ≤8 high.

Item-specific rules. Item 2: consecutive enrollment explicitly stated = 2; not stated = 1. Item 3: prospective = 2; mixed prospective/retrospective = 1; retrospective = 0. Item 5: blinded or independent central review = 2; standardized radiographic criteria without blinding = 1; criteria not reported = 0. Item 6: median follow-up ≥12 months, or all patients followed ≥1 year or until death = 2; median <12 months = 1; not reported = 0. Item 7: loss to follow-up documented <5% = 2; not clearly reported, or 5–20% = 1; >20% non-evaluable or censored before 12 months = 0. Item 8: formal power calculation = 2; prespecified pilot, feasibility, or dose-escalation target = 1; none = 0.

All studies were scored from the full-text article. Weinberg et al. (2026) was scored from the accepted, unedited manuscript.

ᵃ ROADS (Radiation One and Done Study) is a randomized controlled trial. Because only its Cs-131 (tile-based radiation therapy) arm contributed data, it was scored as a single-arm cohort using the eight non-comparative items, for consistency with the other included studies.

**Supplementary Table S3. Sensitivity analyses**

| **Outcome** | **Scenario** | **k (SRS / Cs-131)** | **Pre-operative SRS, % (95% CI); events/n** | **Cs-131 brachytherapy, % (95% CI); events/n** | **Test for subgroup differences p** | **Meta-regression β (95% CI), p** |
| --- | --- | --- | --- | --- | --- | --- |
| 12-month local control | Primary analysis (19 studies) | 14 / 4 | 90 (86–93); 759/853 | 97 (90–99); 206/213 | 0.054 | 1.16 (0.20 to 2.13), 0.019 |
| Leptomeningeal disease | Primary analysis (19 studies) | 14 / 2 | 5.1 (3.3–7.8); 47/880 | 9.1 (5.5–15); 14/154 | 0.088 | 0.55 (−0.19 to 1.29), 0.144 |
| Radiation necrosis | Primary analysis (19 studies) | 12 / 4 | 5.2 (2.9–9.2); 47/791 | 0.7 (<0.1–29); 5/213 | 0.326 | −1.27 (−2.71 to 0.17), 0.084 |
| 12-month local control | Without STEP (18 studies; database search only) | 13 / 4 | 88 (86–90); 698/790 | 97 (90–99); 206/213 | 0.025 | 1.36 (0.57 to 2.14), <0.001 |
| Leptomeningeal disease | Without STEP (18 studies; database search only) | 13 / 2 | 5.8 (4.1–8.2); 47/817 | 9.1 (5.5–15); 14/154 | 0.154 | 0.48 (−0.20 to 1.16), 0.167 |
| Radiation necrosis | Without STEP (18 studies; database search only) | 11 / 4 | 5.0 (2.5–9.6); 43/728 | 0.7 (<0.1–29); 5/213 | 0.342 | −1.30 (−2.86 to 0.26), 0.102 |
| 12-month local control | Wernicke 2017 whole cohort (42 patients / 46 lesions, incl. 10 previously irradiated) | 14 / 4 | 90 (86–93); 759/853 | 97 (90–99); 214/221 | 0.052 | 1.20 (0.23 to 2.17), 0.016 |
| Radiation necrosis | Wernicke 2017 whole cohort (42 patients / 46 lesions, incl. 10 previously irradiated) | 12 / 4 | 5.2 (2.9–9.2); 47/791 | 0.6 (<0.1–30); 5/221 | 0.313 | −1.31 (−2.76 to 0.14), 0.076 |
| 12-month local control | Without the ROADS arm (Weinberg et al) | 14 / 3 | 90 (86–93); 759/853 | 95 (87–98); 104/110 | 0.209 | 0.71 (−0.27 to 1.69), 0.156 |
| Leptomeningeal disease | Without the ROADS arm (Weinberg et al) | 14 / 1 | 5.1 (3.3–7.8); 47/880 | 7.8 (3.0–19); 4/51 | 0.417 | 0.41 (−0.88 to 1.70), 0.536 |
| Radiation necrosis | Without the ROADS arm (Weinberg et al) | 12 / 3 | 5.2 (2.9–9.2); 47/791 | not estimable (0/110 events) | — | — |
| 12-month local control | Without PROPS-BM (Prabhu et al) | 13 / 4 | 91 (86–94); 393/437 | 97 (90–99); 206/213 | 0.077 | 1.11 (0.05 to 2.16), 0.040 |
| Leptomeningeal disease | Without PROPS-BM (Prabhu et al) | 13 / 2 | 4.9 (2.7–8.6); 28/476 | 9.1 (5.5–15); 14/154 | 0.111 | 0.53 (−0.36 to 1.41), 0.246 |
| Radiation necrosis | Without PROPS-BM (Prabhu et al) | 11 / 4 | 4.8 (2.3–9.7); 22/375 | 0.7 (<0.1–29); 5/213 | 0.349 | −1.31 (−2.92 to 0.30), 0.110 |
| Radiation necrosis | Without Kite et al | 12 / 3 | 5.2 (2.9–9.2); 47/791 | 2.9 (0.4–18); 5/162 | 0.562 | −0.92 (−2.34 to 0.50), 0.206 |
| 12-month local control | Inverse variance (REML, Hartung–Knapp) in place of GLMM | 14 / 4 | 88 (85–90); 759/853 | 96 (77–99); 206/213 | 0.066 | 0.86 (0.09 to 1.64), 0.029 |
| Leptomeningeal disease | Inverse variance (REML, Hartung–Knapp) in place of GLMM | 14 / 2 | 6.4 (4.3–9.4); 47/880 | 9.1 (0.3–78); 14/154 | 0.256 | 0.37 (−0.40 to 1.14), 0.346 |
| Radiation necrosis | Inverse variance (REML, Hartung–Knapp) in place of GLMM | 12 / 4 | 6.6 (3.7–12); 47/791 | 3.4 (0.8–13); 5/213 | 0.191 | −0.85 (−2.09 to 0.39), 0.177 |
| 12-month local control | Studies with reported median follow-up ≥12 months | 8 / 2 | 90 (84–93); 573/644 | 97 (88–99); 149/154 | 0.101 | 1.20 (0.08 to 2.32), 0.036 |
| 12-month local control | Crude counts where reported in place of rate-derived counts | 14 / 4 | 90 (86–93); 759/853 | 98 (95–99); 209/213 | 0.001 | 1.79 (0.69 to 2.90), 0.001 |
| 12-month local control | Observed-count studies only | 5 / 2 | 90 (81–94); 165/185 | 99 (95–100); 140/141 | 0.009 | 2.82 (0.77 to 4.87), 0.007 |
| 12-month local control | Patient-level denominators only | 12 / 3 | 91 (86–94); 381/424 | 96 (88–99); 168/175 | 0.168 | 0.84 (−0.27 to 1.95), 0.136 |
| Radiation necrosis | Patient-level denominators only | 11 / 3 | 4.8 (2.3–9.7); 22/375 | 1.8 (0.1–22); 5/175 | 0.478 | −1.02 (−2.62 to 0.59), 0.215 |
| 12-month local control | Leave-one-out: without Agrawal | 13 / 4 | 90 (85–93); 715/806 | 97 (90–99); 206/213 | 0.047 | 1.20 (0.22 to 2.18), 0.016 |
| 12-month local control | Leave-one-out: without Biau | 13 / 4 | 88 (86–90); 698/790 | 97 (90–99); 206/213 | 0.025 | 1.36 (0.57 to 2.14), <0.001 |
| 12-month local control | Leave-one-out: without Deguchi | 13 / 4 | 90 (86–93); 740/833 | 97 (90–99); 206/213 | 0.048 | 1.19 (0.22 to 2.17), 0.016 |
| 12-month local control | Leave-one-out: without Higazy | 13 / 4 | 91 (86–94); 730/818 | 97 (90–99); 206/213 | 0.070 | 1.10 (0.10 to 2.10), 0.031 |
| 12-month local control | Leave-one-out: without Hoyle | 13 / 4 | 91 (86–94); 677/757 | 97 (90–99); 206/213 | 0.075 | 1.09 (0.07 to 2.11), 0.036 |
| 12-month local control | Leave-one-out: without Jansen | 13 / 4 | 89 (86–91); 738/832 | 97 (90–99); 206/213 | 0.029 | 1.24 (0.32 to 2.16), 0.008 |
| 12-month local control | Leave-one-out: without Kutuk | 13 / 4 | 90 (85–93); 723/814 | 97 (90–99); 206/213 | 0.053 | 1.18 (0.18 to 2.18), 0.020 |
| 12-month local control | Leave-one-out: without Li | 13 / 4 | 90 (86–93); 738/829 | 97 (90–99); 206/213 | 0.062 | 1.14 (0.13 to 2.15), 0.027 |
| 12-month local control | Leave-one-out: without Mallela | 13 / 4 | 90 (86–94); 739/827 | 97 (90–99); 206/213 | 0.068 | 1.09 (0.13 to 2.04), 0.026 |
| 12-month local control | Leave-one-out: without Marwaha | 13 / 4 | 89 (86–91); 729/822 | 97 (90–99); 206/213 | 0.029 | 1.23 (0.29 to 2.17), 0.010 |
| 12-month local control | Leave-one-out: without Prabhu | 13 / 4 | 91 (86–94); 393/437 | 97 (90–99); 206/213 | 0.077 | 1.11 (0.05 to 2.16), 0.040 |
| 12-month local control | Leave-one-out: without Rogers | 13 / 4 | 90 (86–93); 754/847 | 97 (90–99); 206/213 | 0.058 | 1.15 (0.16 to 2.13), 0.022 |
| 12-month local control | Leave-one-out: without Schröder | 13 / 4 | 90 (86–93); 747/840 | 97 (90–99); 206/213 | 0.053 | 1.17 (0.19 to 2.16), 0.020 |
| 12-month local control | Leave-one-out: without Yaprak | 13 / 4 | 90 (86–93); 746/837 | 97 (90–99); 206/213 | 0.065 | 1.12 (0.12 to 2.11), 0.028 |
| 12-month local control | Leave-one-out: without Kite | 14 / 3 | 90 (86–93); 759/853 | 98 (91–100); 159/162 | 0.042 | 1.76 (0.46 to 3.06), 0.008 |
| 12-month local control | Leave-one-out: without Mahapatra | 14 / 3 | 90 (86–93); 759/853 | 98 (90–100); 187/192 | 0.059 | 1.45 (0.35 to 2.55), 0.010 |
| 12-month local control | Leave-one-out: without Weinberg | 14 / 3 | 90 (86–93); 759/853 | 95 (87–98); 104/110 | 0.209 | 0.71 (−0.27 to 1.69), 0.156 |
| 12-month local control | Leave-one-out: without Wernicke | 14 / 3 | 90 (86–93); 759/853 | 96 (88–99); 168/175 | 0.120 | 0.91 (−0.09 to 1.91), 0.074 |

Each row repeats the primary binomial-normal GLMM analysis after the stated change; the inverse-variance rows use REML with the Hartung–Knapp adjustment. Rows with no events in one arm are not estimable and are reported as counts. Leave-one-out analyses are shown for the primary endpoint (12-month local control).

**Supplementary Table S3 (continued). Twelve-month local control within pre-operative SRS by fractionation**

| **Comparison** | **Group** | **Studies (k)** | **Controlled/n** | **Pooled LC, % (95% CI)** | **Test for subgroup differences p** |
| --- | --- | --- | --- | --- | --- |
| Single vs hypofractionated (studies with one regimen) | Single | 7 | 214/250 | 86 (81–89) | 0.005 |
| Single vs hypofractionated (studies with one regimen) | Hypofractionated | 3 | 110/114 | 96 (91–99) | 0.005 |
| Single (incl. mixed schedules: Jansen, Prabhu) vs hypofractionated | Single | 9 | 601/687 | 87 (85–90) | 0.009 |
| Single (incl. mixed schedules: Jansen, Prabhu) vs hypofractionated | Hypofractionated | 3 | 110/114 | 96 (91–99) | 0.009 |

**Supplementary Table S4. Study-level outcome data and derivation of 12-month counts**

| **Arm** | **Study** | **12-month LC, controlled/n** | **LC count derivation** | **Unit** | **Median follow-up, mo** | **Follow-up ≥12 mo** | **LMD, events/n** | **RN, events/n** | **Median OS, mo** | **Fractionation** | **Notes** |
| --- | --- | --- | --- | --- | --- | --- | --- | --- | --- | --- | --- |
| Pre-operative SRS | Agrawal 2024 | 44/47 | 12-month Kaplan–Meier estimate × n | patients | 14.68 | Yes | 3/29 | 8/32 | 17.6 | Single | LMD 3/29 and RN 8/32: the paper reports LMD and RN per visit among patients with data; 3/29 is the minimum cumulative LMD count (6-month visit); 8 RN in total (Methods), all after 6 months, among the 32 followed beyond 6 months. LC 44/47 from the 12-month Kaplan–Meier estimate read from Figure 3. |
| Pre-operative SRS | Biau 2026 (STEP) | 61/63 | 12-month Kaplan–Meier estimate × n | patients | 12.7 | Yes | 0/63 | 4/63 | NR | Hypofractionated | Identified through follow-up of a trial protocol found by the search (published 30 Oct 2026, after the database search). KPS reported only in bands (90–100 in 64.2%). OS median not reached (ITT 67). |
| Pre-operative SRS | Deguchi 2022 | 19/20 | Reported 12-month rate × n | patients | NR | No | 1/20 | 0/20 | 8.4 | Hypofractionated |  |
| Pre-operative SRS | Higazy 2025 | 29/35 | Reported 12-month rate × n | patients | 11.8 | No | 1/35 | 3/35 | 13.8 | Single | LMD counts classic LMD only (1/35); a pachymeningeal case at 11 months is not counted. |
| Pre-operative SRS | Hoyle 2025 | 82/96 | Observed count over follow-up | patients | 9 | No | 11/96 | 2/96 | 12.8 | Single | Denominator 96 as printed with the local-failure result (14 of 96) is used for all Hoyle outcomes; the preoperative cohort is given as 95 elsewhere in the paper. LC 82/96 is the crude count. RN 2/96, both in the phase 1 component. |
| Pre-operative SRS | Jansen 2024 | 21/21 | Reported 12-month rate × n | patients | 10.32 | No | 1/21 | 1/21 | NR | Mixed | Age reported only as means by arm (NR here). Dose 16 Gy is the single-fraction median (15 patients); 6 patients had 24–27 Gy in 3 fractions (pairing inferred from per-arm counts). RN from the 4-month adverse-event table. |
| Pre-operative SRS | Kutuk 2024 | 36/39 | Observed count over follow-up | patients | 14 | Yes | 4/39 | 2/39 | NR | Not classified | Patient-level numbers at risk (39) analyzed; the source reports LF, LMD and RN per lesion (47). |
| Pre-operative SRS | Li 2022 | 21/24 | Reported 12-month rate × n | patients | 16.5 | Yes | 0/24 | NR | 26.4 | Single |  |
| Pre-operative SRS | Mallela 2024 | 20/26 | Reported 12-month rate × n | patients | NR | Yes | 1/26 | 1/16 | 17 | Single | Age reported only as a mean (NR here). |
| Pre-operative SRS | Marwaha 2025 | 30/31 | Observed count over follow-up | patients | 9 | No | 3/31 | 0/31 | 15 | Hypofractionated |  |
| Pre-operative SRS | Prabhu 2023 | 366/416 | Reported 12-month rate × n | lesions | 18.1 | Yes | 19/404 | 25/416 | 17.2 | Mixed |  |
| Pre-operative SRS | Rogers 2024 | 5/6 | Observed count over follow-up | patients | NR | No | 0/6 | 0/6 | 6.3 | Single | 13 enrolled; 12 received pre-operative SRS and resection (1 went straight to surgery). OS reported for all 13. |
| Pre-operative SRS | Schröder 2025 | 12/13 | Observed count over follow-up | lesions | 13.8 | Yes | NR | NR | NR | Not classified | 12 patients / 13 resected brain metastases (one resected tumour was a WHO grade 4 astrocytoma; 2 patients declined surgery). LC 12/13 per lesion, observed. LMD and RN NR: only 6-month rates reported. Fractionation not classified: 5 of 16 tumours had an undescribed schedule. |
| Pre-operative SRS | Yaprak 2025 | 13/16 | 12-month Kaplan–Meier estimate × n | patients | 15 | Yes | 0/16 | 1/16 | NR | Single | 18 resected index lesions; 3 non-index lesions had SRS alone and are not counted. |
| Pre-operative SRS | Yoo 2025 | NR | Not reported | — | NR | No | 3/70 | NR | 9.1 | Single |  |
| Cs-131 | Kite 2026 | 47/51 | 12-month Kaplan–Meier estimate × n | patients | 12.4 | Yes | 4/51 | 0/51 | 11 | N/A | RN 0/51 confirmed from registry data by study investigators; not reported in the publication. Dose '60 Gy at 5 mm' is the GammaTile standard prescription, not stated in the paper (BED10 ~90 Gy at the cavity wall). |
| Cs-131 | Mahapatra 2025 | 19/21 | 12-month Kaplan–Meier estimate × n | patients | 2.93 | No | NR | 0/21 | 5.9 | N/A |  |
| Cs-131 | Weinberg 2026 | 102/103 | Observed count over follow-up | patients | 13.3 | Yes | 10/103 | 5/103 | 42.5 | N/A | Systemic therapy 48.5% = before screening (Supplement Table S5). LMD 10/103 from Supplement Table S12. Dose '60 Gy at 5 mm' is the GammaTile standard prescription, not stated in the paper. |
| Cs-131 | Wernicke 2017 | 38/38 | Observed count over follow-up | lesions | 11.9 | No | NR | 0/38 | 20.1 | N/A |  |

Wernicke et al. (2017): treatment-naïve stratum (32 patients, 38 lesions); the whole cohort (42 patients, 46 lesions) is a sensitivity analysis (Table S3). Follow-up ≥12 mo: median follow-up of at least 12 months, or all patients followed for at least 1 year. Reported rate or Kaplan–Meier estimate × n: published 12-month estimate multiplied by the analyzed sample size and rounded; these treat all patients as followed to 12 months and overstate precision.

**Supplementary Table S5. PRISMA 2020 checklist**

| **Section and topic** | **Item** | **Checklist item** | **Location where item is reported** |
| --- | --- | --- | --- |
| Title | 1 | Identify the report as a systematic review | Title page |
| Abstract | 2 | Abstract (PRISMA 2020 for Abstracts) | Abstract (structured) |
| Introduction | 3 | Rationale | Introduction |
|  | 4 | Objectives | Introduction, final paragraph |
| Methods | 5 | Eligibility criteria | Methods, Protocol and Eligibility Criteria |
|  | 6 | Information sources | Methods, Search Strategy; Supplementary Table S1 (databases, dates; one study by other methods) |
|  | 7 | Search strategy | Supplementary Table S1 |
|  | 8 | Selection process | Methods, Study Selection and Data Extraction |
|  | 9 | Data collection process | Methods, Study Selection and Data Extraction |
|  | 10a | Data items: outcomes | Methods, Outcomes and Derivation of Event Counts |
|  | 10b | Data items: other variables | Methods, Study Selection and Data Extraction |
|  | 11 | Study risk of bias assessment | Methods, Risk of Bias; Supplementary Table S2 |
|  | 12 | Effect measures | Methods, Outcomes and Derivation of Event Counts; Statistical Analysis |
|  | 13a | Synthesis methods: eligibility for each synthesis | Methods, Outcomes and Derivation of Event Counts |
|  | 13b | Synthesis methods: data preparation | Methods, Outcomes and Derivation of Event Counts; Supplementary Table S4 |
|  | 13c | Synthesis methods: tabulation and visual display | Figures 2–3; Table 3 |
|  | 13d | Synthesis methods: methods and rationale | Methods, Statistical Analysis |
|  | 13e | Synthesis methods: exploring heterogeneity | Methods, Statistical Analysis (modality; fractionation within pre-operative SRS) |
|  | 13f | Synthesis methods: sensitivity analyses | Methods, Statistical Analysis; Supplementary Table S3; Supplementary Figures S1–S3 |
|  | 14 | Reporting bias assessment | Methods, Statistical Analysis (not assessed: four brachytherapy studies) |
|  | 15 | Certainty assessment | Not performed |
| Results | 16a | Study selection | Results, Study Selection; Figure 1 |
|  | 16b | Studies excluded at full text, with reasons | Results, Study Selection; Figure 1; Supplementary Table S6 |
|  | 17 | Study characteristics | Results, Study Characteristics; Tables 1–2 |
|  | 18 | Risk of bias in studies | Results, Risk of Bias; Supplementary Table S2 |
|  | 19 | Results of individual studies | Figures 2–3; Supplementary Table S4 |
|  | 20a | Results of syntheses: characteristics and risk of bias of contributing studies | Results, Study Characteristics and Risk of Bias |
|  | 20b | Results of syntheses: statistical syntheses | Results, Local Control; Leptomeningeal Disease and Radiation Necrosis; Overall Survival; Table 3 |
|  | 20c | Results of syntheses: investigations of heterogeneity | Results, Local Control; Supplementary Table S3 |
|  | 20d | Results of syntheses: sensitivity analyses | Results, Local Control; Supplementary Table S3; Supplementary Figures S1–S3 |
|  | 21 | Reporting biases | Not assessed (Methods, Statistical Analysis) |
|  | 22 | Certainty of evidence | Not assessed |
| Discussion | 23a–d | Interpretation, limitations of the evidence and of the review, implications | Discussion; Limitations; Future Directions |
| Other information | 24a–c | Registration and protocol | Methods, Protocol and Eligibility Criteria; Statements and Declarations (not registered) |
|  | 25 | Support | Statements and Declarations: Funding |
|  | 26 | Competing interests | Statements and Declarations: Competing interests |
|  | 27 | Availability of data, code, and other materials | Statements and Declarations: Data and code availability; Supplementary Table S4 |

**Supplementary Table S6. Reports assessed in full text and excluded, with reasons**

| **Report** | **Title** | **DOI** | **Reason** | **Detail** |
| --- | --- | --- | --- | --- |
| Bugarini 2023 | Pre-operative stereotactic radiosurgery for cerebral metastatic disease: A retrospective dose-volume study | 10.1016/j.radonc.2022.07.019 | Intervention | Pre-operative plans were hypothetical; all patients received post-operative SRS |
| Yeboa 2025 | Therapy, Safety, and Logistics of Preoperative vs Postoperative Stereotactic Radiation Therapy: A Preliminary Analysis of a Randomized Clinical Trial | 10.1001/jamaoncol.2025.1770 | Outcome | 30-day outcomes only; no local control, LMD or RN |
| Asher 2014 | A New Treatment Paradigm: Neoadjuvant Radiosurgery Before Surgical Resection of Brain Metastases With Analysis of Local Tumor Recurrence | 10.1016/j.ijrobp.2013.12.013 | Overlapping cohort | Levine Cancer Institute patients, a PROPS-BM site |
| Bander 2023 | Safety and efficacy of Cesium-131 brachytherapy for brain tumors | 10.1007/s11060-023-04352-0 | Overlapping cohort | Weill Cornell Cs-131 series; the most recent report (Wernicke 2017) was retained |
| Chuck 2026 | Intraoperative, interstitial brachytherapy in newly diagnosed brain metastases through cesium-131 collagen tile implant: A multi-institutional case series | 10.1093/noajnl/vdag113 | Overlapping cohort | Same registry (STaRT) as Kite 2026 |
| Julie 2021 | A matched-pair analysis of clinical outcomes after intracavitary cesium-131 brachytherapy versus stereotactic radiosurgery for resected brain metastases | 10.3171/2020.3.JNS193419 | Overlapping cohort | Weill Cornell Cs-131 series; the most recent report (Wernicke 2017) was retained |
| Murphy 2024 | Phase I trial of dose escalation for preoperative stereotactic radiosurgery for patients with large brain metastases | 10.1093/neuonc/noae076 | Overlapping cohort | Cleveland Clinic patients, a PROPS-BM site |
| Palmer 2022 | Fractionated pre-operative stereotactic radiotherapy for patients with brain metastases: a multi-institutional analysis | 10.1007/s11060-022-04073-w | Overlapping cohort | Ohio State patients, a PROPS-BM site |
| Patel 2016 | Comparing preoperative with postoperative stereotactic radiosurgery for resectable brain metastases: A multi-institutional analysis | 10.1227/NEU.0000000000001096 | Overlapping cohort | Levine Cancer Institute patients, a PROPS-BM site |
| Patel 2017 | Comparing pre-operative stereotactic radiosurgery (SRS) to post-operative whole brain radiation therapy (WBRT) for resectable brain metastases: a multi-institutional analysis | 10.1007/s11060-016-2334-3 | Overlapping cohort | Levine Cancer Institute patients, a PROPS-BM site |
| Patel 2018 | Neoadjuvant Stereotactic Radiosurgery Before Surgical Resection of Cerebral Metastases | 10.1016/j.wneu.2018.08.107 | Overlapping cohort | Levine Cancer Institute patients, a PROPS-BM site |
| Perlow 2023 | Comparing pre-operative versus post-operative single and multi-fraction stereotactic radiotherapy for patients with resectable brain metastases | 10.1016/j.ctro.2022.11.004 | Overlapping cohort | Ohio State patients, a PROPS-BM site |
| Perlow 2026 | Preoperative Single-Fraction Versus Fractionated Stereotactic Radiation Therapy: A Single-Institutional Analysis of 299 Resected Metastases | 10.1016/j.adro.2026.102118 | Overlapping cohort | Ohio State patients, a PROPS-BM site |
| Perlow 2026 | Preoperative Versus Postoperative Fractionated Stereotactic Radiation Therapy: A Single-Institution Analysis of 534 Resected Metastases | 10.1016/j.ijrobp.2025.08.010 | Overlapping cohort | Ohio State patients, a PROPS-BM site |
| Pham 2016 | Neurocognitive function and quality of life in patients with newly diagnosed brain metastasis after treatment with intra-operative cesium-131 brachytherapy: a prospective trial | 10.1007/s11060-015-2009-5 | Overlapping cohort | Weill Cornell Cs-131 series; the most recent report (Wernicke 2017) was retained |
| Prabhu 2019 | Preoperative stereotactic radiosurgery before planned resection of brain metastases: Updated analysis of efficacy and toxicity of a novel treatment paradigm | 10.3171/2018.7.JNS181293 | Overlapping cohort | Levine Cancer Institute patients, a PROPS-BM site |
| Prabhu 2021 | Preoperative Radiosurgery for Resected Brain Metastases: The PROPS-BM Multicenter Cohort Study | 10.1016/j.ijrobp.2021.05.124 | Overlapping cohort | Same patients as PROPS-BM (Prabhu 2023), the pre-operative SRS anchor cohort |
| Prabhu 2024 | Single-Fraction Versus Fractionated Preoperative Radiosurgery for Resected Brain Metastases: A PROPS-BM International Multicenter Cohort Study | 10.1016/j.ijrobp.2023.09.012 | Overlapping cohort | Same patients as PROPS-BM (Prabhu 2023), the pre-operative SRS anchor cohort |
| Prabhu 2025 | Standard Dose Versus Reduced Dose Single Fraction Preoperative Radiosurgery for Resected Brain Metastases (PROPS-BM) International Multicenter Cohort Study | 10.1016/j.adro.2025.101794 | Overlapping cohort | Same patients as PROPS-BM (Prabhu 2023), the pre-operative SRS anchor cohort |
| Udovicich 2022 | From Postoperative to Preoperative: A Case Series of Hypofractionated and Single-Fraction Neoadjuvant Stereotactic Radiosurgery for Brain Metastases | 10.1227/ONS.0000000000000101 | Overlapping cohort | Peter MacCallum Cancer Centre patients, a PROPS-BM site |
| Udovicich 2025 | International collaboration of neoadjuvant stereotactic radiosurgery for brain metastases: The INTERNEO individual patient data pooled analysis | 10.1016/j.radonc.2024.110641 | Overlapping cohort | Pooled individual-patient analysis; replaced by its non-overlapping sites (Li, Rogers, Yoo, Schröder) |
| Wernicke 2014 | Phase I/II study of resection and intraoperative cesium-131 radioisotope brachytherapy in patients with newly diagnosed brain metastases | 10.3171/2014.3.JNS131140 | Overlapping cohort | Weill Cornell Cs-131 series; the most recent report (Wernicke 2017) was retained |
| Wernicke 2016 | Surgical technique and clinically relevant resection cavity dynamics following implantation of cesium-131 brachytherapy in patients with brain metastases | 10.1227/NEU.0000000000000986 | Overlapping cohort | Weill Cornell Cs-131 series; the most recent report (Wernicke 2017) was retained |
| Zeller 2024 | Early experience and perioperative risk of GammaTile for upfront brain metastases: Report from a prospective multicenter study | 10.1093/noajnl/vdae156 | Overlapping cohort | Same registry (STaRT) as Kite 2026 |
| Dharnipragada 2023 | GammaTile (GT) as a brachytherapy platform for rapidly growing brain metastasis | 10.1093/noajnl/vdad062 | Population | Newly diagnosed and recurrent lesions mixed; newly diagnosed outcomes not reported separately |
| Nakaji 2020 | Resection and Surgically Targeted Radiation Therapy for the Treatment of Larger Recurrent or Newly Diagnosed Brain Metastasis: Results From a Prospective Trial | 10.7759/cureus.11570 | Population | Newly diagnosed and recurrent lesions mixed; newly diagnosed outcomes not reported separately |
| Vetlova 2017 | Preoperative Stereotactic Radiosurgery of Brain Metastases: Preliminary Results | 10.7759/cureus.1987 | Population | 8 of 19 patients treated for recurrence; newly diagnosed outcomes not reported separately |
| Xia 2018 | Outcomes of Metastatic Brain Lesions Treated with Radioactive Cs-131 Seeds after Surgery: Experience from One Institution | 10.7759/cureus.3075 | Population | Lesions previously irradiated |

28 reports excluded: overlapping cohort 22; population 4; intervention 1; outcome 1. When reports shared patients, one non-overlapping set of cohorts was retained (Methods, Study Selection and Data Extraction).

**Supplementary Figure S1. Twelve-month local control, inverse-variance pooling (REML, Hartung–Knapp).**

**Supplementary Figure S2. Leptomeningeal disease, inverse-variance pooling (REML, Hartung–Knapp).**

**Supplementary Figure S3. Radiation necrosis, inverse-variance pooling (REML, Hartung–Knapp).**
